# Statement of Retraction: Bicomponent polymeric micelles for pH-controlled delivery of doxorubicin

**DOI:** 10.1080/10717544.2024.2355035

**Published:** 2024-06-10

The Editors and Publisher of the journal Drug Delivery, have retracted the following article:

Chunyun Wang, Peilan Qi, Yan Lu, Lei Liu, Yanan Zhang, Qianli Sheng, Tianshun Wang, Mengying Zhang, Rui Wang & Shiyong Song (2020) Bicomponent polymeric micelles for pH-controlled delivery of doxorubicin, Drug Delivery, 27:1, 344–357, DOI: 10.1080/10717544.2020.1726526

Since publication, concerns have been raised about the integrity of the data in the article. When approached for an explanation, the authors have been unable to fully address the concerns raised. As verifying the validity of published work is core to the integrity of the scholarly record, we are therefore retracting the article. All authors listed in this publication have been informed.

We have been informed in our decision-making by our policy on publishing ethics and integrity and the COPE guidelines.

The retracted article will remain online to maintain the scholarly record, but it will be digitally watermarked on each page as ‘Retracted’.

